# Evaluation of the Short-Term Music Therapy on Brain Functions of Preterm Infants Using Functional Near-Infrared Spectroscopy

**DOI:** 10.3389/fneur.2021.649340

**Published:** 2021-09-28

**Authors:** Haoran Ren, Liangyan Zou, Laishuan Wang, Chunmei Lu, Yafei Yuan, Chenyun Dai, Wei Chen

**Affiliations:** ^1^School of Information Science and Technology, The Center for Intelligent Medical Electronics, Fudan University, Shanghai, China; ^2^Department of Neonatology, Children's Hospital of Fudan University, Shanghai, China; ^3^Shanghai Key Laboratory of Medical Imaging Computing and Computer Assisted Intervention, Shanghai, China

**Keywords:** preterm infants, short-term music therapy, functional near-infrared spectroscopy, resting-state functional connectivity, Mozart music

## Abstract

Music contains substantial contents that humans can perceive and thus has the capability to evoke positive emotions. Even though neonatal intensive care units (NICUs) can provide preterm infants a developmental environment, they still cannot fully simulate the environment in the womb. The reduced maternal care would increase stress levels in premature infants. Fortunately, music intervention has been proved that it can improve the NICU environment, such as stabilize the heart rate and the respiratory rate, reduce the incidence of apnea, and improve feeding. However, the effects of music therapy on the brain development of preterm infants need to be further investigated. In this paper, we evaluated the influence of short-term music therapy on the brain functions of preterm infants measured by functional near-infrared spectroscopy (fNIRS). We began by investigating how premature babies perceive structural information of music by calculating the correlations between music features and fNIRS signals. Then, the influences of short-term music therapy on brain functions were evaluated by comparing the resting-state functional connectivity before and after the short-term music therapy. The results show that distinct brain regions are responsible for processing corresponding musical features, indicating that preterm infants have the capability to process the complex musical content. However, the results of network analysis show that short-term music intervention is insufficient to cause the changes in cerebral functional connectivity. Therefore, long-term music therapy may be required to achieve the deserved effects on brain functional connectivity.

## Introduction

Music contains substantial contents that humans can perceive, such as the variations of loudness, timbre, and rhythm. Processing of music is a complex process, requiring different brain regions to participate in multilevel processing (1). The investigation of the brain in response to different sound categories is fundamental to exploring the functional organization of the auditory cortex (2). Due to the multidimensional characteristics of music, it can evoke positive emotions (3). Therefore, there have been a lot of studies on music therapy, exploring the effects of music on psychological, behavioral, and physiological factors (4–6).

Generally, preterm infants have to suffer the pain from pathologic and clinical cares, such as intestinal colic and infection, and also to endure ambient noise, such as random noise generated by instruments or light. Worse still, reduced maternal cares would increase stress levels in premature infants (7). In addition, ambient noise has a negative impact on the development of preterm infants (8). Therefore, it is an urgent need for premature infants to reduce the impact of external noise in NICUs on neonatal brain functions. Previous studies have provided the evidence that music can improve the environment of NICUs, such as stabilize the heart rate and the respiratory rate, reduce the incidence of apnea, and improve feeding (9–11). However, current knowledge about the processing of music information in preterm infants and the effects of music therapy on the brain function of preterm infants is sparse, requiring further systematic studies (12).

Scientific evidences and neuroimaging studies have supported that newborns have the ability to process music in the early stage (12, 13). The ability of sensing and learning music in newborns may stem from the third trimester of gestation. The music and speech exposed to the fetus in the womb provide the basis for the later training of music and speech (14). Studies of neonatal behaviors and physiological signals demonstrate that musical perception exists during fetal life (15, 16). It has shown a decreased heart rate in the newborns who were exposed to music training during prenatal life when the newborns listened to familiar music excerpts (15). The specific brain networks of preterm infants who received a period time of music therapy would be active when exposed to the familiar music, indicating the ability of processing the temporal features and specific tempos (3). Combined with the above studies, premature babies have the ability to perceive and remember sound information. More importantly, music training would enhance the ability of temporal structure processing both in music and speech in 9-month age infants (17). Therefore, reducing negative external stimulation of premature infants is beneficial to the normal development of brain functions in premature infants, and music therapy has profound effects on the brain development of premature babies.

An adult study has shown that a larger scale of a brain network would be involved to process the naturalistic stimulus compared with artificial manipulated music. It proved that the study under ecological background can more accurately and completely reflect the neural circuits of the brain in music processing (18). However, there is still a lack of research on how premature babies process structural information of continuous music. In addition, it should be noted that although preterm and full-term infants can perceive music or speech information, the results of studies on the hemispheric lateralization in newborns processing music are not consistent (19–21). A right hemispheric lateralization in response to constant music stimulus was observed in newborns, while a left lateralized activation, implicated in the inferior frontal cortex and limbic structure, was shown in the newborns when exposed to altered music (19). However, two other studies investigated music processing in newborns and showed no lateralization using fMRI and fNIRS (20, 21). Above all, those studies still lack a systematic study on how preterm infants process musical information during music therapy and evaluation of the effects of short-term music therapy on premature brain functions.

fNIRS uses optical signals to measure changes in the hemoglobin concentration of the cerebral cortex to assess brain activation (22). Compared with other neuroimaging techniques, such as EEG, fMRI, and PET, fNIRS has the advantages of relatively high spatial and temporal resolution, better comfort, and less strict requirements on the experimental environment. It is more suitable for research in special populations and natural states. Research on fNIRS has grown exponentially in the last 20 years (23). Among the many significant fNIRS studies, one of the major contributions is the study of brain development. These studies shed light on cognitive processes in the developing brain that cannot be explored by other neuroimaging techniques, such as the cognitive processing of vision, sensory, and smell in infants, as well as the processing of language and sound (24). It provides a new method and important scientific values for the study of neural development.

This paper explores the short-term effects of music therapy on the brain functions of premature infants using fNIRS from two aspects. Firstly, we focus on the brain functional response to the continuous musical stimuli in preterm infants using fNIRS. Musical features, representing the characteristics of rhythm, timbre, tone, and dynamic, were extracted. Second, a correlation analysis between the musical features and the time series of the fNIRS signals was performed to localize the specific regions that participated in the music perception. In light of previous studies (18), we hypothesized that distinct cerebral regions would be involved in processing the different musical features. Additionally, the changes of functional networks were evaluated by resting-state functional connectivity of fNIRS. The channels covering temporal and frontal regions were selected as two regions of interest (ROIs), which were associated with the perception of music and processing musical stimulus (1, 18, 25, 26). The intrahemispheric and interhemispheric connectivities were analyzed to explore the effect of short-term music therapy on the interactions across brain regions. The brain specialization for music processing in preterm infants and the outcomes of short-term music therapy were systematically studied.

## Materials and Methods

### Participants

In this study, preterm infants were recruited from the Neonatal Intensive Care Unit (NICU) in a children hospital affiliated with Fudan University, Shanghai, China. Before the experiment, preterm infants were screened by experienced neonatologists based on inclusion and exclusion criteria. The details of the inclusion and exclusion criteria have been introduced in our previous work (27). The approval of this study was received from the Ethics Committee of Children's Hospital of Fudan University, Shanghai, China (2017-235). Written informed consents were obtained from their parents.

The study is designed as a prospective, randomized, controlled clinical trial. A total of 40 preterm infants (gestation age = 34.2 ± 1.12 weeks, 22 boys, 18 girls) were randomized to either the music therapy group (MTG) or the control group (CG). The median chronological age at the time of inclusion was 10 days (range from 3 to 15), and the clinical condition of the preterm infants was stable. A total of 20 preterm infants, who received the common clinical care without music intervention, were randomized to the CG. The experiment was conducted once a day for three consecutive days. The specific intervention procedures were introduced in the following Music intervention section. Seven (in the MTG) and nine (in the CG) subjects failed to complete 3 days' intervention due to physiological diseases or discharge from hospital. Additionally, 3 out of 13 subjects in the MTG were discarded due to the obvious motion artifacts. Two participants in the CG were excluded from the data due to obvious motion artifacts. The remaining 20 infants were included for further analysis: 10 MTG and 10 CG. The preterm infants included in this experiment (both the MTG and the CG) received standard of care.

### Music Intervention

The experiment was started after the subjects began receiving formula feeding. The preterm infants were positioned supine in the incubators. The MTG listened to classical music: *Mozart Sonata for Two Pianos in D Major*, K,448 (performed by Karl Ulrich Schnabel and Helen Schnabel, duration: 1,242 s). Previous studies have shown that Mozart K.448 had effects on spatial task performance and positive effects to reduce the occurrence of seizures (27–29). Additionally, in our previous study, the comparisons between the experimental and control groups in the post resting state using sample entropy have proved that Mozart K.448 has the capability to calm preterm infants (27). Therefore, Mozart K.448 was selected as a mean of musical intervention in this study. In order to prevent auditory fatigue, we manually selected a 180-s musical segment, which contained the complete music information.

The intervention was conducted for three successive days. The whole procedure for each day lasted for 44 min, containing a 10-min pre-resting state, four repetitions of 3-min musical stimulation with a 3-min rest between two consecutive trials, and a 10-min post-resting state. The music was played by a headphone (SONY MDR-Z7, Japan). During the experiment, environmental noise was reduced as much as possible to ensure that the background noise in the incubator was below 40 dB. The sound pressure level of the music near the infants' ears was below 65 dB. The CG had the same handling as the MTG, but the headphones near the subjects' ears were on mute.

### fNIRS Acquisition

**Figure 1:**
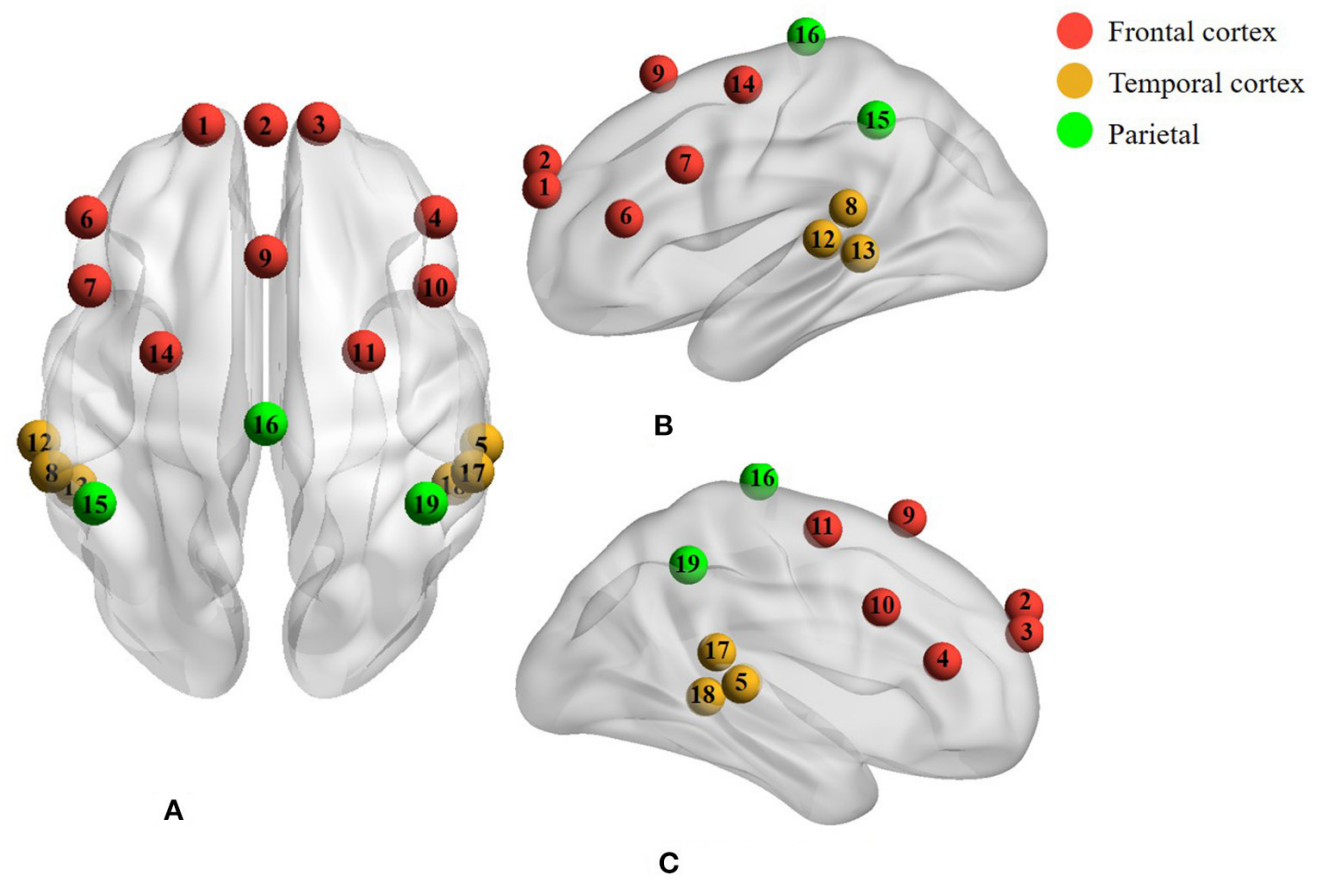
A total of 19 channels covered the cerebral cortex of the frontal, temporal, and parietal regions with the red, yellow, and green colors, respectively. **(A)** Axial view. **(B)** Left lateral view. **(C)** Right lateral view.

The cerebral hemodynamic changes in response to music stimulus were collected by the NIRScout measurement system (NIRx Medical Technologies, LLC., USA), which was based on a continuous wave technique with eight LED sources and eight detectors at wavelengths of 760 and 850 nm. In this study, seven sources and seven detectors were placed in frontal, temporal, and parietal regions according to the international 10-10 system, forming 19 channels in total. The distribution of fNIRS electrodes is shown in Figure 1. The distance between the source and the detector was 3 cm. The sampling rate of fNIRS was automatically set to 8.9286 Hz. Dim light was guaranteed using opaque cloths covering the incubators as the fNIRS signals were susceptible to lights. Music and trigger signals were controlled by a presentation software package (E-Prime 3, Psychology Software Tools, Sharpsburg, PA). Trigger signals were sent to fNIRS using a trigger cable.

### NIRS Preprocessing

The received attenuated light signals were converted into relative changes of oxygen hemoglobin (HbO2) and de-oxygen hemoglobin (HHb) concentration according to the modified Beer–Lambert Law using the nirsLAB software (NIRx Medical Technologies, USA). Considering the characteristics of age dependence of differential path length factors (DPFs), the parameters of DPFs were set to 5.286 (at a wavelength of 760) and 4.2238 (at a wavelength of 850) (30, 31). In this study, only the changes of HbO2 were recruited due to its pronounced changes in response to brain activities.

**Figure 2:**
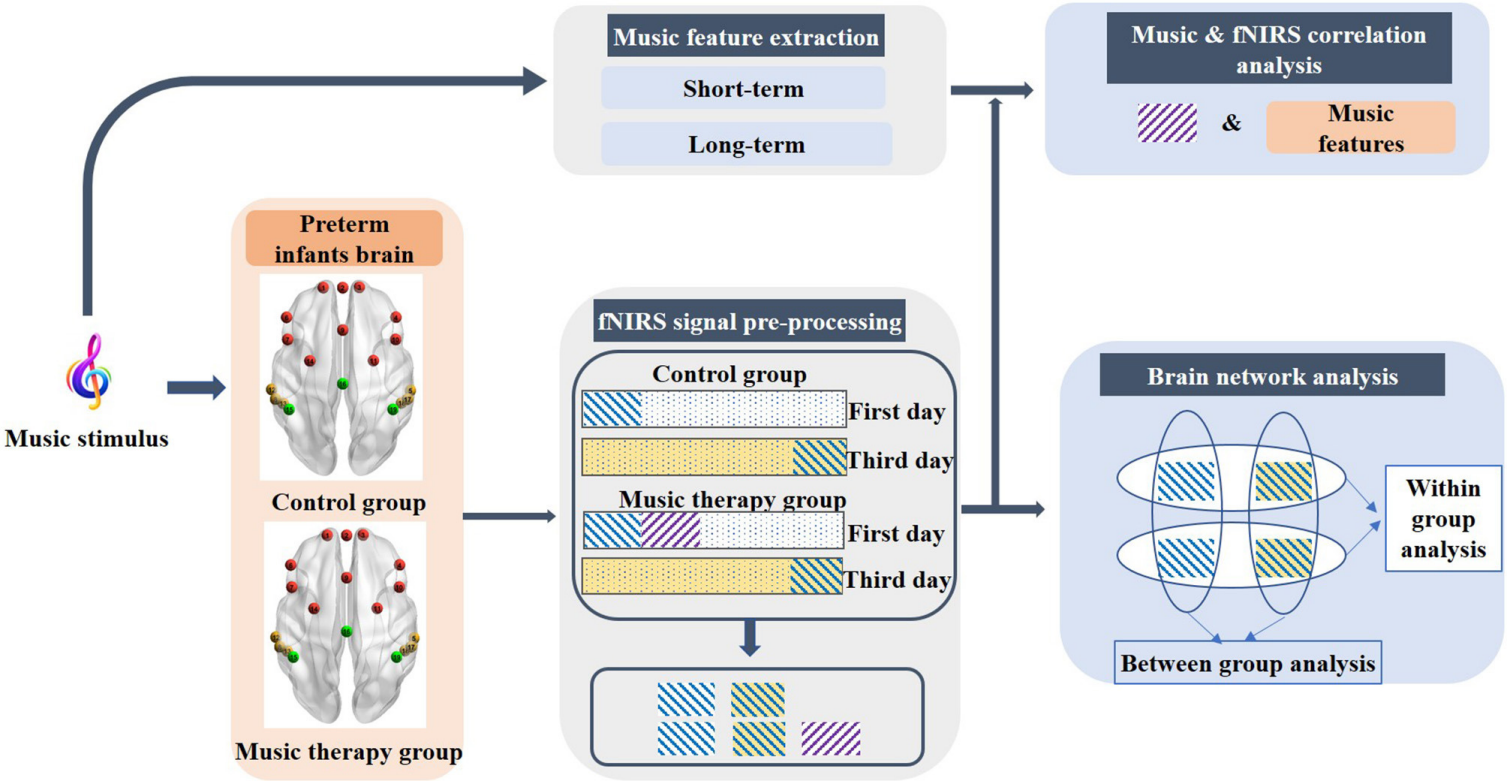
Block diagram of the data acquisition and analysis. The purple rectangle with diagonal stripes represents the first sessions of the fNIRS signal during the music stimulus on the first day. The blue diagonal stripe rectangular with white and light-yellow background represents pre-resting state on the first day and the post-resting state on the third day, respectively.

In general, the changes in HbO2 in the human brain cortex are contaminated by nature oscillations of cardiac activity, respiratory information, and Mayer wave (32). Thus, a band-pass filter (0.01–0.3 Hz) was applied to eliminate the low-frequency drift and high-frequency physiological interferences. Following this, an elimination step of motion artifacts was conducted to remove the spikes stemming from movements using moving standard deviation and spline interpolation (33). In this study, we focus on two aspects: the cortex structures related to various music component stimulus, which was evaluated by the correlations between musical features and fNIRS signals, and the effect of short-term music therapy on cerebral functional connectivity evaluated by brain network analysis. The block diagram of data acquisition and analysis is shown in Figure 2.

### Correlation Between Music and fNIRS

In this part, we focused on the investigation of the cortex structures related to various components of the musical stimulus. The brain activation information was obtained by calculating the correlation between fNIRS signals that underwent a music stimulus and the various musical features. According to previous adult studies that investigated the intersubject correlations when different subjects experienced a common file stimulus, a significant reduction of correlation was observed during the second viewing of the film (34). Therefore, the first session of the fNIRS signal on the first day of premature infants was selected for further analysis. The length of the fNIRS signals was the same as the duration of the music (3 min). The corresponding fNIRS signals were the purple rectangle with diagonal stripes as shown in Figure 2.

Then, 24 features were extracted through the MIRToolbox (35), reflecting the timbral, tonal, and rhythmic aspects of the music (see Supplementary A). Two categories of musical features, i.e., short-term features and long-term features, were extracted underlying the properties of music. Specifically, the short-term features consisted of zero crossing rate, spectral centroid, spectral spread, spectral roll-off, spectral entropy, spectral flatness, roughness, and sub-band flux (10 sub-band components) representing timbral, dynamical, and other components of music (18). The long-term features were comprised of pulse clarity, fluctuation centroid, mode, and key clarity, expressing the rhythmic and tonal components of music. The short- and long-term features were extracted through a 25-ms and 1-s window, respectively, which was the empirical window length representing the corresponding information of music (35, 36). After getting the time series of musical features, all the time series of musical features were resampled to the same length of fNIRS signals. Considering that the fNIRS signal is based on the neurovascular coupling mechanism, it has a time delay in response to the stimulus (37). The resampled time series of musical features were then convolved with double-gamma hemodynamic response function (HRF) (38).

In order to obtain the cortical structure involving the processing of distinct music components, correlation analysis was performed between the time series of individual music components and the time series of the fNIRS signal using Pearson analysis (39). For each subject, the Pearson correlation coefficients between each individual music component and all the 19 channels representing different brain regions were arranged into a 19^*^19 r map. The individual r map should be transformed into a group map, since the objective of this study was to explore the intersubject correlations between musical features and fNIRS signals. We employed the method of group analysis utilized in a previous study (18). Firstly, the individual r maps were converted to Z-score maps using Fisher's Z transformation and subsequently normalized by the correction factor $1/\sqrt{df-3}$, where *df* represented the effective degrees of freedom (see Supplementary B).

Following this, the normalized Z-score maps were converted to p-maps. The group maps were then created using the Fisher's *p*-value technique, which was calculated by the formula shown in Eq. 1 (38):


(1) $$\begin{array}{r} T=\;-2\overset{k}{\underset{i=1}{\sum }}\log p_{i} \end{array}$$

where k represented the k subjects in the experiment (k = 10), and the T-statistic was calculated at each channel independently. The T-statistic was distributed as a chi-square distribution with 2k degrees of freedom under the null hypothesis of no activation in the specific brain region. Hence, the large values of T ($\chi_{0.01}^{2}\left(20\right)\;=\;37.57$) lead to the rejection of the null hypothesis (38).

### Network Analysis

In order to evaluate the effects of short-term music therapy on resting-state functional connectivity, we compared the intrahemispheric and interhemispheric connectivity on the intragroup level and the intergroup level from two aspects (40), containing the left–right correlations and hemispheric functional connectivity. Three minutes of the fNIRS signals in the pre-resting state on the first day and the post-resting state on the third day was selected for comparisons, which was shown as the blue diagonal stripe rectangular with white and light-yellow background in Figure 2. Previous studies on music therapy have established the findings that brain regions involved in music therapy are implicated in frontal regions, the auditory cortex, the sensorimotor, and supplementary areas. The frontal lobe and the temporal lobe were selected as regions of interest (ROI) in this study, that is, left-frontal (L-F), right-frontal (R-F), left-temporal (L-T), and right-temporal (R-T). Due to the limited number of channels, the frontal region consisted of four channels, and the temporal region was comprised of three channels in each hemisphere.

Firstly, the left–right correlations distributed on both hemispheres, as one of the interhemispheric connectivity, were calculated for each ROI. The correlation values were used as a criterion to evaluate the connectivity strength between the left and right hemispheres. Specifically, for an individual subject, Pearson correlation was used to calculate the correlation coefficients between symmetrical channels in the left and right hemispheres of each ROI, such as channels 1 and 3 in the forehead, and channels 5 and 12 in the temporal lobes as distributed in Figure 1. Therefore, four and three paired channels were contained in the frontal and temporal regions, respectively. Following this, the Fisher's r-z was used to convert the correlation coefficient r values to *z*-values. The averaged *z*-values across subjects were further converted to *r*-values by Fisher's z-r. The *r*-values were ultimately utilized as the correlation values in the specific ROI. The correlation values were compared between the MTG and the CG, as well as within each group.

**Figure 3:**
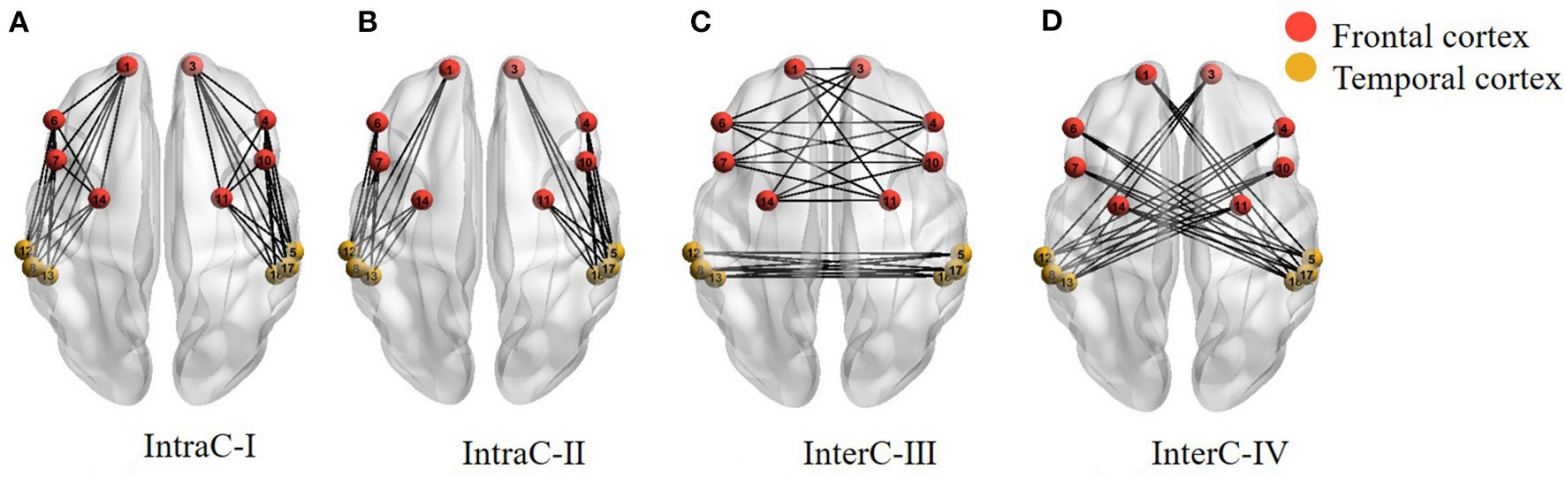
Four categories of the brain networks representing intra- and inter-hemispheric functional connectivity. **(A)** IntraC-I: connectivity within one hemisphere; **(B)** IntraC-II: connectivity between two different ROIs within one hemisphere; **(C)** InterC-III: connectivity between symmetrical ROIs in the two hemispheres; **(D)** InterC-IV: connectivity between asymmetrical ROIs in the two hemispheres.

Secondly, nodes (fNIRS channels) and edges (connection between channels) formed the basis of brain networks. The hemispheric functional connectivity was assessed through the averaged edge numbers as the following four categories: connectivity within one hemisphere (IntraC-I), connectivity between two different ROIs within one hemisphere (IntraC-II), connectivity between symmetrical ROIs in the two hemispheres (InterC-III), and connectivity between asymmetrical ROIs in the two hemispheres (InterC-IV). The diagram of the brain networks is shown as Figure 3. Before calculating the numbers of edges, the correlation matrixes were obtained by computing the Pearson correlation coefficients. IntraC-I and IntraC-II were the intrahemispheric connectivity containing left hemispheric and right hemispheric connectivity. InterC-III and InterC-IV were the interhemispheric connectivity between symmetrical ROIs and asymmetrical ROIs, respectively. InterC-III was comprised of two clusters: frontal InterC-III and temporal InterC-III. In each cluster, both the symmetrical and asymmetrical channel pairs were calculated between the symmetrical ROIs, corresponding to Figure 3C, for instance, the symmetrical channel pairs of 1 and 3, and the asymmetrical channel pairs of 1 and 4 in frontal-InterC-III. Similarly, InterC-IV was grouped by the two connectivity clusters: L-F and R-T, and L-T and R-F. After getting the above four categories of correlation matrixes, the number of edges in the networks was calculated through different threshold values. The correlation matrixes were converted into a binary graph according to the threshold. The edges larger than the threshold were set to 1 and all others were set to 0. A sparsity range of threshold 0.4–0.9 with the interval of 0.05 was selected. Lastly, the edge numbers of the four categories of correlation matrixes were obtained with different thresholds.

### Statistical Analysis

The difference of left–right correlations between the two groups was investigated by conducting Kruskal-Wallis test, and the Wilcoxon signed rank test was performed within each group. In order to compare the differences in the edges between the MTG and the CG, one-way repeated measures ANOVA was performed. *Post hoc* analyses were conducted with Bonferroni correction if necessary. The numbers of threshold (11 levels) were defined as the repeated factor. A significance level of *p* < 0.05 was used. Mauchyly's test of sphericity was utilized to assess the statistical assumption of sphericity. The Greenhouse–Geisser correction was conducted when necessary. All statistical analyses were conducted in SPSS (version 20, IBM Corp., Armonk, NY).

## Results

A total of 20 preterm infants were included in this study, with 10 subjects in the MTG and 10 subjects in the CG. A total of 4 trials were performed daily for 3 days, with a total of 12 trials per subject. Statistical analysis on the demographic characteristics of the two groups was performed, including gestation age, birth weight, 1- and 5-min Apgar scores, and postnatal age and weight at the day of study. There was no significant difference between the two groups (see Table 1).

**Table 1:** Demographic characteristics of the study groups using a significant level *p* < 0.05.

**Clinical parameters**	**Group**		***P*-value**
	**MTG** **(*n* = 10)**	**CG** **(*n* = 10)**	
GA (week)	34.5 ± 1.00	33.8 ± 0.78	0.207
BW	2231 ± 331	1995 ± 267	0.116
1-min Apgar score	9.2 ± 0.42	8.6 ± 1.43	0.239
5-min Apgar score	9.5 ± 0.52	9.1 ± 0.88	0.223
PA	10.2 ± 2.89	8.2 ± 3.82	0.535

*MTG, music therapy group; CG, control group; GA, gestation age; BW, birth weight; PA, postnatal age*.

### Perception of Music Components

We began by investigating the relationship between the music components and the fNIRS signals when preterm infants were listening to the music. The results of the cortical regions representing significant activation during the perception of different music components are shown in Table 2. The left and right hemispheres activated by different acoustic components, and the corresponding Brodmann area (BA) was listed in detail.

**Table 2:** Activated cerebral regions evaluated by correlation results between musical features and fNIRS signals.

**Musical feature**	**Left hemisphere**	**BA**	**chan**	**T-statistic**	**Right hemisphere**	**BA**	**chan**	**T-statistic**
**Timbre**								
Zero crossing	Medial to Superior temporal gyrus—Transverse temporal gyrus of Heschl	42	8	41.21				
Roughness	Posterior part of superior temporal gyrus	22	13	46.51				
Centroid	Inferior frontal gyrus	45	6	43.78				
Roll off	Inferior frontal gyrus	45	6	54.86				
Entropy	Prefrontal cortex	10	1	41.07	Prefrontal cortex	10	3	41.58
	Inferior frontal gyrus	44	7	50.32	Inferior frontal gyrus	45	4	40.34
	Posterior part of superior temporal gyrus	22	13	41.97				
Flatness	Inferior frontal gyrus	45	6	40.85	Posterior part of superior temporal gyrus	22	5	45.34
Spread	-	-	-	-	-	-	-	-
Sub-band 1	Posterior part of superior temporal gyrus	22	13	46.12	Postcentral gyrus	2	16	41.45
	Postcentral gyrus	2	16	41.45				
Sub-band 2	Posterior part of superior temporal gyrus	22	13	43.50	Postcentral gyrus	2	16	41.96
	Postcentral gyrus	2	16	41.96				
Sub-band 3	Postcentral gyrus	2	16	48.24	Postcentral gyrus	2	16	48.24
Sub-band 4	Superior frontal gyrus	6	14	41.29	Postcentral gyrus	2	16	62.80
	Postcentral gyrus	2	16	62.80				
Sub-band 5	-	-	-		-	-	-	
Sub-band 6	Middle frontal gyrus	8	9	42.10	Middle frontal gyrus	8	9	42.10
	Posterior part of superior temporal gyrus	22	13	41.81				
Sub-band 7	Middle frontal gyrus	8	9	42.27	Middle frontal gyrus	8	9	42.27
Sub-band 8	Posterior part of superior temporal gyrus	22	13	43.34				
Sub-band 9	Posterior part of superior temporal gyrus	22	13	46.28	Postcentral gyrus	2	16	42.58
	Postcentral gyrus	2	16	42.58				
Sub-band 10	Posterior part of superior temporal gyrus	22	13	46.70	Postcentral gyrus	2	16	43.33
	Postcentral gyrus	2	16	43.33				
**Others**								
Spectral flux	Posterior part of superior temporal gyrus	22	13	46.41	Postcentral gyrus	2	16	45.01
	Postcentral gyrus	2	16	45.01				
**Dynamic/loudness**								
RMS	Posterior part of superior temporal gyrus	22	13	46.00	Postcentral gyrus	2	16	41.70
	Postcentral gyrus	2	16	41.70				
**Tonal**								
Mode	-	-	-					
Key clarity	Prefrontal cortex	10	2	43.43	Prefrontal cortex	10	2	43.43
		44	7	46.17				
**Rhythm**								
Pulse clarity	Posterior part of superior temporal gyrus	22	13	42.86	Postcentral gyrus	2	16	48.80
	Superior frontal gyrus	6	14	43.77				
	Postcentral gyrus	2	16	48.80				
Event density	-	-	-	-	-	-	-	-
Metroid	-	-	-	-	-	-	-	-

*T-statistic is distributed as a chi-square distribution with a significant level p <0.01 ($\chi_{0.01}^{2}\left(20\right)=37.57$). Large values of T correspond to the activated channels*.

*BA, Brodmann area; chan, channel*.

As for the timbral components, the sensory areas (BA 22 and 42) including the superior temporal gyrus and Heschl's gyrus activated by roughness (*T* = 46.51, *p* < 0.01) and spectral entropy (*T* = 41.97, *p* < 0.01), and Broca's area (BA: 45) activated by spectral centroid (T = 43.78, *p* < 0.01), roll-off (*T* = 54.86, *p* < 0.01), and flatness (*T* = 40.85, *p* < 0.01), showed a left hemispheric lateralization. Additionally, the timbral features would increase the neural activation in the bilateral prefrontal cortex (BA 10) activated by spectral entropy (left hemisphere: *T* = 41.07, *p* < 0.01; right hemisphere: *T* = 41.58, *p* < 0.01), bilateral middle frontal (BA 8) activated by sub-band 6 (*T* = 41.81), sub-band 8 (*T* = 42.27, *p* < 0.01), and bilateral postcentral gyrus (BA 2) activated by sub-band 1 (*T* = 41.45, *p* < 0.01), sub-band 2 (*T* = 41.96, *p* < 0.01), sub-band 3 (*T* = 48.24, *p* < 0.01), and sub-band 4 (*T* = 62.80, *p* < 0.01). The dynamic component was associated with the activation in the left superior temporal gyrus (BA 22, *T* = 46.00, *p* < 0.01) and the bilateral postcentral gyrus (BA 2, *T* = 41.70, *p* < 0.01). The tonal component would activate the bilateral prefrontal cortex (BA: 10, *T* = 43.43, *p* < 0.01). The rhythm components showed activations in left hemispheric lateralization containing the superior temporal gyrus (BA 22) activated by pulse clarity (*T* = 42.86, *p* < 0.01), as well as the superior frontal gyrus (BA 6) activated by pulse clarity (*T* = 43.77, *p* < 0.01). Apart from the left hemispheric lateralization, the activation in the bilateral postcentral gyrus (BA 2, *T* = 48.80, *p* < 0.01) was also observed.

Therefore, multiple brain regions are involved in processing structural information of music. Processing of musical components involves not only the activation of the left auditory cortex but also the frontal and postcentral gyrus.

### Resting-State Functional Connectivity

**Figure 4:**
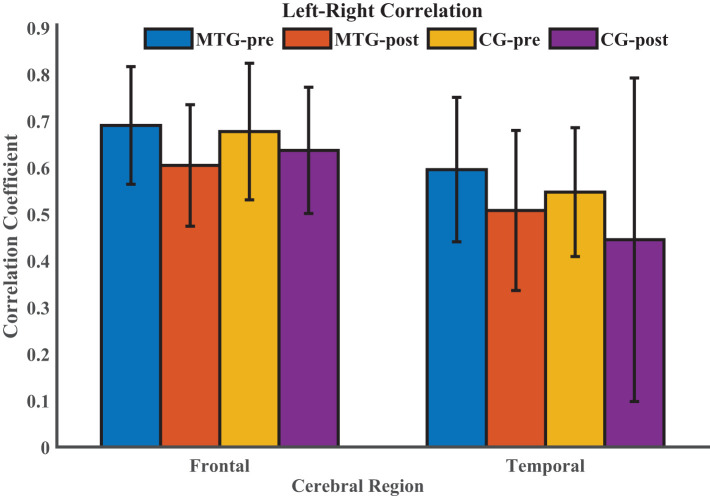
Comparison of left–right correlation values between the MTG and the CG in each ROI. MTG-pre: resting state before music therapy in the music therapy group; MTG-post: resting state after music therapy in the music therapy group; CG-pre: resting state before music therapy in the control group; CG-post: resting state after music therapy in the control group.

Then, we explored the changes of intrahemispheric and interhemispheric connectivity induced by the short-term music therapy by conducting the resting-state function connectivity. The comparison of left–right correlation values between the MTG and the CG in each ROI is shown in Figure 4. Kruskal-Wallis test was performed in frontal and temporal regions to compare the difference in left–right correlation strength between the MTG and the CG. However, there was no significant difference between the two groups [frontal: *F*(1, 18) = 0.46, *p* = 0.496; temporal: *F*(1, 18) = 0.01, *p* = 0.940]. Additionally, Wilcoxon signed rank test was performed within each group to evaluate the effect of short-term music therapy on the left–right correlation strength in the MTG (*p* = 0.131), and to evaluate the changes of the left–right correlation strength in the courses of short-term brain development in the CG (*p* = 0.232).

**Figure 5:**
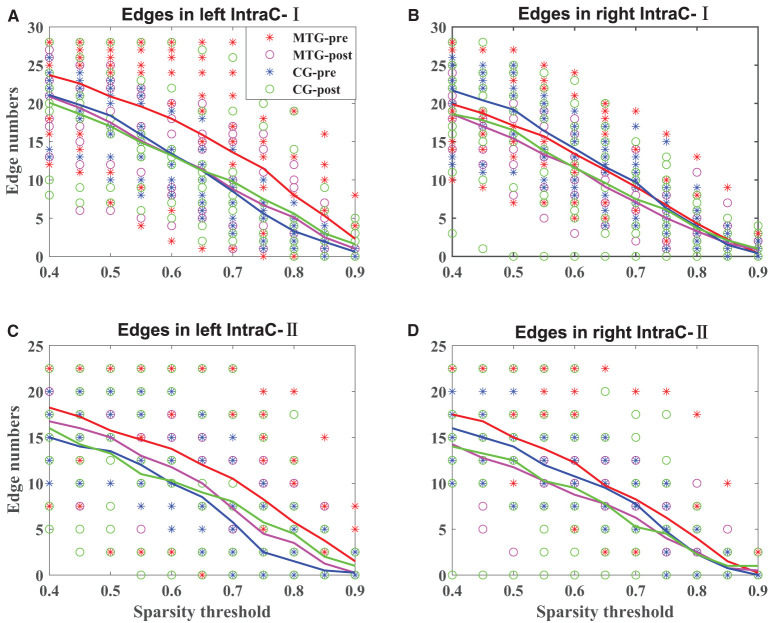
Intrahemispheric connectivity in the MTG and the CG. The solid lines in red, magenta, blue, and green represent the averaged numbers of resting-state functional connectivity of pre-music therapy in the MTG, post-music therapy in the MTG, pre-music therapy in the CG, and post-music therapy in the CG, respectively. The asterisks and hollow circles represent the values of an individual subject. **(A,B)** Represents the number of intrahemispheric connectivity within the left and right hemispheres under different thresholds in IntraC-I, respectively. **(C,D)** Represents the number of intrahemispheric connectivity between different ROIs in the left and right hemispheres under different thresholds in IntraC-II, respectively.

**Figure 6:**
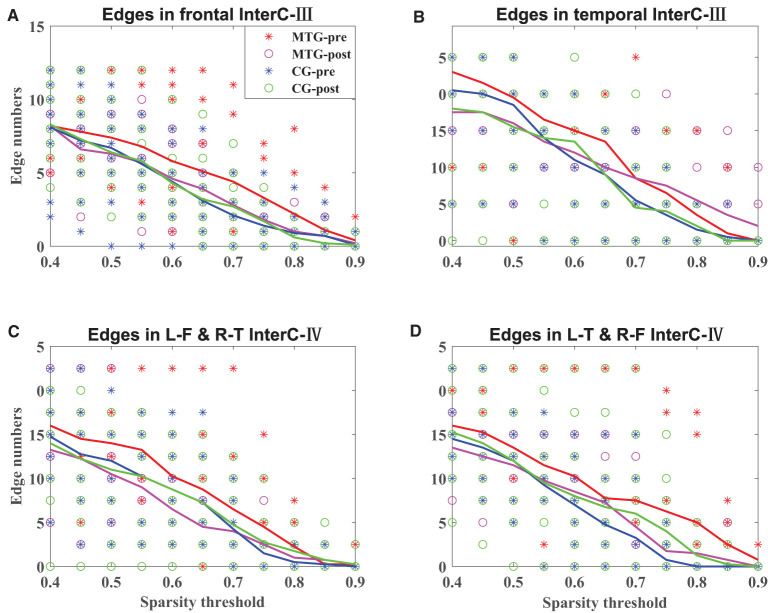
Interhemispheric connectivity in the MTG and the CG. The solid lines in red, magenta, blue, and green represent the averaged numbers of resting-state functional connectivity of pre-music therapy in the MTG, post-music therapy in the MTG, pre-music therapy in the CG, and post-music therapy in the CG, respectively. The asterisks and hollow circles represent the values of an individual subject. **(A,B)** Represents the number of symmetrical interhemispheric connectivity in frontal and temporal regions in InterC-III, respectively. **(C,D)** Represents the number of interhemispheric connectivity between frontal and temporal regions in InterC-IV, respectively.

The results of the intrahemispheric and interhemispheric connectivity evaluated by the numbers of edges are shown in Figures 5, 6, respectively. For the investigation of intrahemispheric connectivity, the functional connectivity during pre-resting state in IntraC-I between the MTG and the CG (MTG-pre and CG-pre) has been evaluated before comparing the difference of functional connectivity induced by music therapy. The statistical analysis results are shown in Table 3. And there was no significant difference between the two groups before music therapy [left hemisphere: *F*(1, 18) = 2.403, *p* = 0.139; right hemisphere: *F*(1, 18) = 0.107, *p* = 0.747). Figures 5A,B represents the number of functional connections of the left and right hemispheres under different thresholds in IntraC-I, respectively. Statistical analysis was performed between groups (MTG-post and CG-post) and within groups (MTG-pre and MTG-post and CG-pre and CG-post). However, no significant difference was found in the comparison of between groups [left hemisphere: *F*(1, 18) = 0.033, *p* = 0.937; right hemisphere: *F*(1, 18) = 0.062, *p* = 0.806] and within groups [MTG-left: *F*(1, 18) = 1.729, *p* = 0.205; MTG-right: *F*(1, 18) = 0.677, *p* = 0.421; CG-left: *F*(1, 18) = 0.016, *p* = 0.900; CG-right: *F*(1, 18) = 0.622, *p* = 0.441]. Similarly, the left IntraC-II and right IntraC-II were analyzed, and the results are shown in Figures 5C,D. Comparisons between groups (MTG-post and CG-post) and within groups (MTG-pre and MTG-post and CG-pre and CG-post) were conducted. There was no significant difference between groups [left hemisphere: *F*(1, 18) = 0.039, *p* = 0.854, right hemisphere: *F*(1, 18) = 0.007, *p* = 0.934] and within groups [MTG-left: *F*(1, 18) = 0.857, *p* = 0.367; MTG-right: *F*(1, 18) = 1.737, *p* = 0.204; CG-left: *F*(1, 18) = 0.282, *p* = 0.602; CG-right: *F*(1, 18) = 0.627, *p* = 0.439]. We also compared the functional connectivity between the two hemispheres, and no significant difference was shown between them. The procedures of statistical analysis about the interhemispheric connectivity in InterC-III and InterC-IV were the same as those we described above. The results of the interhemispheric connectivity are shown in Figure 6. In InterC-III, there was no significant difference in the comparison between groups [frontal: *F*(1, 18) = 0.070, *p* = 0.795; temporal: *F*(1, 18) = 0.285, *p* = 0.600] and within groups [frontal-MTG: *F*(1, 18) = 0.853, *p* = 0.368; temporal-MTG: *F*(1, 18) = 0.323, *p* = 0.577; frontal-CG: *F*(1, 18) = 0.056, *p* = 0.815; temporal-CG: *F*(1, 18) = 0.357, *p* = 0.558]. In InterC-IV, there was no significant difference in the comparisons between groups [L-F and R-T: *F*(1, 18) = 0.183, *p* = 0.674; L-T and R-F: *F*(1, 18) = 0.006, *p* = 0.941] and within groups in MTG [L-F and R-T: *F*(1, 18) = 1.276, *p* = 0.273; L-T and R-F: *F*(1, 18) = 1.692, *p* = 0.210] and in CG [L-F and R-T: *F*(1, 18) = 0.001, *p* = 0.979; L-T and R-F: *F*(1, 18) = 0.218, *p* = 0.646].

**Table 3:** Comparisons of the intrahemispheric and interhemispheric connectivity between and within groups using a significant level of *p* < 0.05.

**Groups**	**IntraC-I**				**IntraC-II**			
	**Left hemisphere**		**Right hemisphere**		**Left hemisphere**		**Right hemisphere**	
	***F* **(1, 18)****	***P*-value**	***F* **(1, 18)****	***P*-value**	***F* **(1, 18)****	***P*-value**	***F* **(1, 18)****	***P*-value**
MTG-post and CG-post	0.033	0.927	0.062	0.806	0.039	0.854	0.007	0.934
MTG-pre and MTG-post	1.729	0.205	0.677	0.421	0.857	0.367	1.737	0.204
CG-pre and CG-post	0.016	0.900	0.622	0.441	0.282	0.602	0.627	0.439
**Groups**	**InterC-III**				**InterC-IV**			
	**Frontal**		**Temporal**		**L-F and R-T**		**L-T and R-F**	
	***F*** **(1, 18)**	***P*** **-value**	***F*** **(1, 18)**	***P*** **-value**	***F*** **(1, 18)**	***P*** **-value**	***F*** **(1, 18)**	***P*** **-value**
MTG-post and CG-post	0.070	0.795	0.323	0.577	0.183	0.674	0.006	0.941
MTG-pre and MTG-post	0.853	0.368	0.323	0.577	1.276	0.273	1.692	0.210
CG-pre and CG-post	0.056	0.815	0.357	0.558	0.001	0.979	0.218	0.646

*MTG, music therapy group; CG, control group; -pre, before music therapy, -post, after music therapy; L-F and R-T, connectivity between left frontal and right temporal areas; L-T and R-F, connectivity between left temporal and right temporal areas*.

## Discussion

In this study, the cerebral hemodynamics in response to Mozart music and the effects of the short-term music therapy on the brain functions have been investigated by analyzing the correlations between fNIRS signals and music components, and by exploring the resting-state functional connectivity. We first employed the brain regions that were responsible for processing different musical features. The characteristics of timbre, rhythm, dynamic, and tone were extracted and then convolved with the double-gamma HRF. Following this, Pearson correlation analysis was performed between fNIRS signals and music components. The association between cerebral hemodynamics and music in preterm infants was obtained from the group level. Secondly, the effects of the short-term music therapy on the resting-state functional connectivity were investigated. The brain regions involved in music processing, memory, and motor were selected as the ROI, calculating the changes of resting-state functional connectivity before and after music therapy. Two aspects from the left–right correlation to intra- and interhemispheric connectivity were explored.

The brain networks in processing musical features in adults have been well-investigated, while research on musical information processing in preterm infants is scarce. Numerous studies have provided the evidence that the preterm infants are capable of perceiving music and speech. In this study, timbral, dynamic, and rhythmic musical components simultaneously activate the auditory and somatosensory cortices, as well as other areas that contain higher-order cognitive networks, motor cortex, and language regions. Listening to music is a complex process involving multiple sensory responses in the brain (12). The results of this study further demonstrate that preterm infants have the capability of processing musical features, and prove the evidence of synchronous large-scale brain networks involved in musical perception. Consistent with the results of adult studies, timbral components activated the superior temporal gyrus and Heschl's gyrus (18, 41, 42), as well as the left superior frontal gyrus. The activation of the primary somatosensitive cortex, the superior frontal gyrus, and the postcentral gyrus suggests that musical perception could promote motor behaviors. However, the left hemispheric lateralization in the superior temporal gyrus in this study is inconsistent with the right hemispheric lateralization in adults (18). And the left lateralization in the superior temporal gyrus is also shown in processing dynamic and rhythm characteristics. The left lateralization was also shown in previous preterm studies when processing the altered music with key shifting or dissonance (19). The different specialization of the left and right auditory cortices can process acoustic stimuli in terms of temporal and spectrum aspects separately, thus exhibiting right hemisphere laterality to the music stimulus (43, 44). The inconsistent results between our results and previous studies may be caused by the different structures of the music components. Both timbral and tonal components activated the bilateral prefrontal cortex. It has been fully proved in neuroscience research that the activity of the prefrontal cortex is related to high-order cognitive functions, involving working memory, cognitive flexibility, and planning. Therefore, our results consisted with the view that music processing is beneficial to the development of high-order cognitive, socio-emotional, and motor functions of the functional connectivity in premature infants (45). Our results also show that the processing of a rhythmic component is associated with the activation in the auditory cortex, Broca's areas, the premotor cortex, and the supplementary motor cortex. The processing of rhythmic information would improve the development of brain functions in processing language and cognition (17). The synchronically activated auditory cortex, the motor region, and the language region indicates that the processing of rhythmic components can promote the synchronization in breathing, mimicry, and gesture, and synchronization at the neural level (46).

Previous studies have revealed that the influence of music therapy on the brain function of preterm infants is manifested in two aspects: one is the improvement of structural maturation and connection; the other is the enhancement of brain functional connection. Studies on brain functional imaging have shown that music therapy can promote the maturation of micro- and macrostructures and improve the structural connections (45, 47). Music therapy could enhance the functional connectivity in networks implicated in prefrontal regions, the auditory cortex, the sensory motor, and supplementary areas. In addition, a significant increased coupling between salience networks and the networks implicated in superior frontal, auditory, sensorimotor networks, thalamus, and precuneus networks was shown in a resting-state fMRI study (48). Although music contains a wealth of information and the specific music components activate corresponding brain regions, the results of resting-state brain functional connectivity in this study suggest that short-term music therapy is insufficient to cause the changes in cerebral functional connectivity. From the perspective of musical properties, numerous studies have been executed to investigate the effects of Mozart music on task performance or clinical symptoms. However, the attitudes toward the Mozart effect were inconsistent (49). Studies supporting the existence of the Mozart effect have confirmed the significance of the effect through a large number of scientific and clinical studies (49). On the other hand, the studies failed to replicate Rauscher's findings and thus denied the existence of the Mozart effect (49, 50). In order to investigate the influences of Mozart music on cerebral hemodynamics, our previous study has been conducted and has confirmed the capability of the soothing effect of Mozart music on preterm infants (27). Considering the duration of music therapy, the duration of the existing studies was generally more than 2 weeks, while this study only lasted for 3 days. Therefore, we conclude that even if the Mozart effect has a soothing effect on preterm infants, the short-term music intervention is still not enough to have a significant effect on brain functional connectivity (see Supplementary C). Additionally, the development of the cerebral network showed dynamic changes over time from several days to 6 months of postnatal age in infants (45). Previous studies have shown that adequate music therapy could promote the structural maturation of the brain in preterm infants (47), and an enhanced coupling in auditory–sensorimotor was shown in musicians (46, 47). Therefore, based on existing music therapy studies, future work using long-term musical intervention may be necessary in order to achieve positive improvement in brain functions, which will provide insights into the in-depth understanding of the effects of music therapy on brain function in preterm infants.

Although fNIRS has advantages over fMRI during data collection, it has to be noted that the drawbacks of this study are the limited ability of fNIRS to detect the deep brain tissues, such as the basal ganglia detected by fMRI (3), and the limited channels. This leads to an impossible conduction on a comprehensive investigation of the whole brain. Additionally, there is still no standard for music selection in music therapy. In this study, only Mozart music, which is widely used in clinical interventions, was used (28). Music with different characteristics contains different musical information, and the comparison of music therapy with different music types remains to be studied in the future. Because prefrontal areas are activated during music processing, the long-term neurological effects on preterm infants, such as cognitive and memory processing, remain to be further investigated. Although short-term music therapy does not have a significant effect on brain functions, we hypothesized that short-term music therapy may have a potential effect on the future development of infants, so a long-term follow-up of subjects after music therapy will be conducted for future studies. Additionally, since fNIRS sequences do not cover the “whole” brain or deep (but important) structures such as the basal ganglia, the longitudinal analysis (6 or 12 months of age, or with a longer follow-up) for the comparison between (f)MRI and fNIRS, which would provide a comprehensive view in the field of neuroscience, can be further investigated.

## Conclusion

In this study, the brain activity of preterm infants was monitored with fNIRS while listening to Mozart music, which acted as the intervention of music therapy. The intervention procedures lasted for three successive days and 12 min for each day. This paper provides a simple approach to investigate the cortex structures related to various musical component stimuli and found left lateralization in the superior temporal gyrus during processing timbral, dynamic, and rhythmic musical components. Specific brain regions were involved in processing the corresponding musical features, indicating that preterm infants have the capability to process the complex musical content. Additionally, network analysis indicates that the short-term music intervention was insufficient to impact the brain functions in preterm infants. A long-term intervention may be required to achieve the goal of improving functional connectivity in preterm infants.

## Data Availability Statement

The original contributions presented in the study are included in the article/Supplementary Material, further inquiries can be directed to the corresponding author/s.

## Ethics Statement

The studies involving human participants were reviewed and approved by Ethics Committee of Children's Hospital of Fudan University, Shanghai, China. Written informed consent to participate in this study was provided by the participants' legal guardian/next of kin.

## Author Contributions

HR acquired the data, analyzed the results, drafted the manuscript, and plotted the figures. LZ, LW, and CL collected the data. YY, CD, and WC revised the manuscript. All authors contributed to the conception and design of the work, interpretation of the results, and the revision of the manuscript, are accountable for all aspects of the work, and have approved the final version of the manuscript.

## Funding

This work was supported by the Shanghai Municipal Science and Technology Major Project (Grant No. 2017SHZDZX01).

## Conflict of Interest

The authors declare that the research was conducted in the absence of any commercial or financial relationships that could be construed as a potential conflict of interest.

## Publisher's Note

All claims expressed in this article are solely those of the authors and do not necessarily represent those of their affiliated organizations, or those of the publisher, the editors and the reviewers. Any product that may be evaluated in this article, or claim that may be made by its manufacturer, is not guaranteed or endorsed by the publisher.
