# Supplementary material for: Evaluation of the Short-Term Music Therapy on Brain Functions of Preterm Infants Using Functional Near-Infrared Spectroscopy
Source: Front Neurol. 2021 Sep 28;12:649340. doi: 10.3389/fneur.2021.649340 (PMC8505667; doi:10.3389/fneur.2021.649340)
Supplement: Supplementary file 1 [file Presentation_1.pdf]

Supplementary Materials:

Evaluation of the short-term music therapy on brain functions of preterm infants using fNIRS

Haoran Ren[1], Liangyan Zou[2], Laishuan Wang[2*], Chunmei Lu[2], Yafei Yuan[1], Chenyun Dai[1*], Wei Chen[1,3*]

[1] The Center for Intelligent Medical Electronics, School of Information Science and Technology, Fudan University, Shanghai, China.

[2] Department of Neonatology, Children's Hospital of Fudan University, Shanghai, China

[3.] Shanghai Key Laboratory of Medical Imaging Computing and Computer Assisted Intervention, Shanghai, China.

*** Correspondence:**
Laishuan Wang: laishuanwang@163.com
Chenyun Dai: chenyundai@fudan.edu.cn
Wei Chen: w_chen@fudan.edu.cn

Supplementary A

24 musical features representing the timbral, tonal, and rhythmic features were extracted through the toolbox of MIRToolbox using the frame-based analyses (1). The frames have a size of 50 ms with 50% overlap and 1 s with 50% overlap for the short-term and long-term features, respectively.

The detailed definition and calculation methods of each musical feature have been described in the user manual of the MIRToolbox. Therefore, a brief description of the musical features utilized in this study is introduced as follows.

Short-term features:

Zero crossing rate: counts the number of sign changes in the music waveforms.

Roughness: assessed by adding the beating provoked by each couple of energy peaks in the spectrum (2).

Spectral centroid: represents the center of gravity of the magnitude spectrum of the short time Fourier transform.

Spectral roll off: estimates the amount of high frequency contained below 85% of the total energy.

Spectral entropy: the relative Shannon entropy (3) calculated using the equation Eq. S.1

$$H_t = \frac{\sum_{n=1}^{N} A_t[n] log A_t[n]}{log N} \qquad (S.1)$$

where A_t is the amplitude spectrum of audio frame at time t and N is the number of frequency bins in the amplitude spectrum. The relative Shannon entropy indicates whether the spectrum

contains predominant peaks or not. For example, a single sine tone has minimal entropy and white noise maximal.
Spectral flatness: defined as the ratio between the geometric mean to the arithmetic mean.
Spectral spread:
Sub-band flux (10 features in total): a measure of fluctuation of frequency contents in ten octave-scaled sub-bands of the spectrum (Exploring perceptual and acoustic correlates of polyphonic timbre).
Spectral flux: a measure of temporal change in the spectrum, obtained by calculating the Euclidian distance between subsequent window-based amplitude spectra.
Root Mean Square (RMS): represents the instantaneous energy of the signal by calculating the root average of the square of the amplitude.
Mode: strength of major of minor mode
Key clarity: a measure of the tonal clarity.
Pulse clarity: an estimate of clarity of the pulse (4)
Event density: estimates the average frequency of events, i.e., the number of events detected per second.
Metroid: indicates the temporal evolution of the metrical activity expressed in BPM.

Supplementary B

The estimation of $-df$ was introduced in (5). The formula was shown in S.2,

$$\frac{1}{df} \approx \frac{1}{N} + \frac{2}{N} \sum_j \frac{N-j}{N} p_{xx}(j) p_{yy}(j) \quad \text{(S.2)}$$

where p_{xx} and p_{yy} were the normalized autocorrelation of fNIRS, and time series of musical features containing N sampling points over the j range of lags. The maximum lag j was empirically set to N/5 (6).

Supplementary C

In the current research on music therapy, especially for premature infants, there is no standard elaboration on the amount and timing of music intervention. Therefore, we summarized the studies using neuroimaging technologies in term/preterm infants to explore the effects of music therapy on brain function. In the comparison of technology, the previous studies have used (f)MRI technologies, which provide whole brain scan at high spatial resolution, to explore the changes of structural and functional connectivity. However, due to the strict requirements on the experimental environment of fMRI, it was difficult to obtain the scanned data during subject simultaneously listening to music in previous studies. This has limited the research on the auditory brain network during listening to music. In terms of the duration of music therapy, previous studies all lasted more than 2 weeks. And these studies have found that music intervention would improve the structural and functional brain connectivity. In this study, fNIRS technique was used to explore the effect of music therapy on the brain function of premature infants. Compared to previous studies, we defined the 3-day music therapy as a short-term treatment in this study. Additionally, our previous study has found significant lower entropy in the resting-state after music therapy, indicating that the Mozart music could achieve the effect of calmness and relaxed state (27). This

also confirms the existence of the Mozart effect. Although short-term music therapy had no significant effect on brain functional connectivity, we speculated that the existing Mozart effect has potential effects on the brain development of premature infants. This makes it necessary for the studies of long-term music therapy and longitudinal follow-up studies in the future.

Table S1 Summary of the music therapy studies using neuroimaging technologies in term/preterm infants

Year	Technology	Participants (n)	Duration	Main results
2016 (7)	MEG, MRI	9-month-old infants (MTG: 20, CG: 19)	15 min * 12 sessions (4 weeks)	Enhanced temporal structure processing not only in music, but also in speech.
2019 (8)	MRI	Term infants (16), preterm infants (MTG: 14, CG: 15)	8 min * 5 times per week until discharge	Increased coupling between networks: the salience network with the superior frontal, auditory, and sensorimotor networks, and the salience network with the thalamus and precuneus networks.
2020 (9)	(f)MRI	Preterm infants (MTG: 24, CG: 16)	20 min * 8 sessions	Increased structural integration in posterior cingulate cortex;<br>Higher functional integration in predominantly left prefrontal, supplementary motor, and inferior temporal brain regions.
2020 (10)	MRI	Term infants (13), preterm infants (MTG: 10, CG:11)	8 min * 5 times per week until discharge	Improved white matter maturation in acoustic radiations, external capsule/ claustrum/extreme capsule and uncinate fasciculus, as well as larger amygdala volumes.
**This study**	**fNIRS**	**Preterm infants (MTG: 10, CG: 10)**	**3 min * 4 trials * 3 days**	**A left lateralization in superior temporal gyrus during processing timbral, dynamic and rhythmic musical components;**<br>**Short-term music intervention was insufficient to impact the functional connectivity.**

MEG: Magnetoencephalography, MTG: music therapy group, CG: control group, (f)MRI: (functional) magnetic resonance imaging, fNIRS: functional Near-infrared spectroscopy.

Supplementary References:

1. Lartillot O, Toiviainen P. A Matlab Toolbox for Musical Feature Extraction from Audio. (2007)9.

2. Sethares W A. Tuning, timbre, spectrum, scale[M]. Springer Science & Business Media, 2005.

3. Shannon CE. A mathematical theory of communication. *The Bell System Technical Journal* (1948) 27:379–423. doi:10.1002/j.1538-7305.1948.tb01338.x

4. Lartillot O, Eerola T, Toiviainen P, Fornari J. Multi-feature modeling of pulse clarity: Design, validation, and optimization. *International Conference on Music Information Retrieval* (2008)

5. The Statistical Analysis of Functional MRI Data. New York, NY: Springer New York (2008). doi:10.1007/978-0-387-78191-4

6. Pyper BJ, Peterman RM. Comparison of methods to account for autocorrelation in correlation analyses of fish data. (1998)14.

7. Zhao TC, Kuhl PK. Musical intervention enhances infants' neural processing of temporal structure in music and speech. *Proc Natl Acad Sci USA* (2016) 113:5212–5217. doi:10.1073/pnas.1603984113

8. Lordier L, Meskaldji D-E, Grouiller F, Pittet MP, Vollenweider A, Vasung L, Borradori-Tolsa C, Lazeyras F, Grandjean D, Van De Ville D, et al. Music in premature infants enhances high-level cognitive brain networks. *Proc Natl Acad Sci USA* (2019)201817536. doi:10.1073/pnas.1817536116

9. Haslbeck FB, Jakab A, Held U, Bassler D, Bucher H-U, Hagmann C. Creative music therapy to promote brain function and brain structure in preterm infants: A randomized controlled pilot study. *NeuroImage: Clinical* (2020) 25:102171. doi:10.1016/j.nicl.2020.102171

10. Sa de Almeida J, Lordier L, Zollinger B, Kunz N, Bastiani M, Gui L, Adam-Darque A, Borradori-Tolsa C, Lazeyras F, Hüppi PS. Music enhances structural maturation of emotional processing neural pathways in very preterm infants. *NeuroImage* (2020) 207:116391. doi:10.1016/j.neuroimage.2019.116391
